# Molecular speciation of *Plasmodium* and multiplicity of *P*. *falciparum* infection in the Central region of Ghana

**DOI:** 10.1371/journal.pgph.0002718

**Published:** 2024-01-18

**Authors:** Enoch Aninagyei, Dakorah Mavis Puopelle, Isaac Tukwarlba, George Ghartey-Kwansah, Juliana Attoh, Godwin Adzakpah, Desmond Omane Acheampong

**Affiliations:** 1 Department of Biomedical Sciences, School of Basic and Biomedical Sciences, University of Health and Allied Sciences, Ho, Ghana; 2 Department of Biomedical Sciences, School of Allied Health Science, University of Cape Coast, Cape Coast, Ghana; 3 Department of Health Information Management, School of Allied Health Science, University of Cape Coast, Cape Coast, Ghana; University of California Irvine, UNITED STATES

## Abstract

Malaria is endemic in the Central region of Ghana, however, the ecological and the seasonal variations of *Plasmodium* population structure and the intensity of malaria transmission in multiple sites in the region have not been explored. In this cross-sectional study, five districts in the region were involved. The districts were Agona Swedru, Assin Central and Gomoa East (representing the forest zone) and Abura-Asebu-Kwamankese and Cape Coast representing the coastal zone. Systematically, blood samples were collected from patients with malaria. The malaria status was screened with a rapid diagnostic test (RDT) kit (CareStart manufactured by Access Bio in Somerset, USA) and the positive ones confirmed microscopically. Approximately, 200 μL of blood was used to prepare four dried blood spots of 50μL from each microscopy positive sample. The *Plasmodium* genome was sequenced at the Malaria Genome Laboratory (MGL) of Wellcome Sanger Institute (WSI), Hinxton, UK. The single nucleotide polymorphisms (SNPs) in the parasite mitochondria (PfMIT:270) core genome aided the species identification of *Plasmodium*. Subsequently, the complexity of infection (COI) was determined using the complexity of infection likelihood (COIL) computational analysis. In all, 566 microscopy positive samples were sequenced. Of this number, *Plasmodium* genome was detected in 522 (92.2%). However, whole genome sequencing was successful in 409/522 (72.3%) samples. In total, 516/522 (98.8%) of the samples contained *P*. *falciparum* mono-infection while the rest (1.2%) were either *P*. *falciparum/P*. *ovale* (*Pf/Po*) (n = 4, 0.8%) or *P*. *falciparum/P*. *malariae/P*. *vivax* (*Pf/Pm/Pv*) mixed-infection (n = 2, 0.4%). All the four *Pf/Po* infections were identified in samples from the Assin Central municipality whilst the two *Pf/Pm/Pv* triple infections were identified in Abura-Asebu-Kwamankese district and Cape Coast metropolis. Analysis of the 409 successfully sequenced genome yielded between 1–6 *P*. *falciparum* clones per individual infection. The overall mean COI was 1.78±0.92 (95% CI: 1.55–2.00). Among the study districts, the differences in the mean COI between ecological zones (p = 0.0681) and seasons (p = 0.8034) were not significant. However, regression analysis indicated that the transmission of malaria was more than twice among study participants aged 15–19 years (OR = 2.16, p = 0.017) and almost twice among participants aged over 60 years (OR = 1.91, p = 0.021) compared to participants between 20–59 years. Between genders, mean COI was similar except in Gomoa East where females recorded higher values. In conclusion, the study reported, for the first time, *P*. *vivax* in Ghana. Additionally, intense malaria transmission was found to be higher in the 15–19 and > 60 years, compared to other age groups. Therefore, active surveillance for *P*. *vivax* in Ghana and enhanced malaria control measures in the 15–19 year group years and those over 60 years are recommended.

## Background

Despite several national and international efforts to halt the rapid spread of malaria, the disease still persists in many parts of the world [1]. According to the World Health Organization, there were estimated malaria cases of 247 million and 619,000 deaths worldwide, as at 2022 [2]. Countries in sub-Saharan Africa (SSA) contributed about 93% of the cases and 94% of the mortalities [3]. In SSA and elsewhere, malaria endemicity is influenced by changes in the biological, ecological, and climatic factors [4, 5]. The changes in the aforementioned factors control the life span of both vector and parasite and sustain malaria infection even in small eco-climatic scales and between ecotypes [6–10].

In Ghana, *P*. *falciparum* (98%) is the most common cause of malaria with the rest attributable to *P*. *malariae* and *P*. *ovale*, either mono or co-infection with *P*. *falciparum* [11]. Inability to detect the non-falciparum parasites for proper management will contribute to increase local transmission of the parasites, which can eventually lead to an outbreak. The routine techniques for detecting the malaria parasite in Ghana is the microscopy and the malaria rapid diagnostic test (mRDT) kit [12]. To some extent, microscopy and mRDT kits could be used to differentiate parasites species causing malaria. However, in cases of low parasitemia and in co-infection, these routine techniques lack sensitivity to differentiate implicating parasites. However, in research laboratories, advanced molecular methods, especially amplicon sequencing, have been found to provide a better differentiation of the *Plasmodium* genome to identify species variabilities based on polymorphisms in the regions of the parasite mitochondria (PfMIT:270) [13]. These single nucleotide polymorphisms (SNPs) in the regions of the mitochondria have made it possible to differentiate *P*. *falciparum* from *P*. *vivax*, *P*. *knowlesi*, *P*. *malariae* and *P*. *ovale* in human samples. In this study, Sanger’s 101 SNPs-barcode was used to identify the malaria parasites. The whole genome sequencing approach used in this study also made it possible to determine the number of genetically distinct clones that was responsible for the malaria. The distinct clones of parasites present in one individual is known as complexity of infection (COI) [14]. It is an important malaria epidemiology indicator since it tells the intensity of malaria transmission in a locality. In the case of *P*. *falciparum*, multiclonal infection is attributable to independent bites of mosquitoes carrying the sporozoites or a single mosquito bite carrying a genetically diverse sporozoite inoculum [15]. The direct relationship between mean COI values and transmission of malaria has been reported [16].

The districts in the Central region of Ghana are either forested or coastal. Irrespective of the ecological settings, the districts experience more rainy months than non-rainy months. It remain unexplored the impact of the differences in ecological settings and seasonal variations on *Plasmodium* population structure and the intensity of the transmissibility of *Plasmodium* spp in these area. Therefore, the objectives of this study was to determine the impact of ecological and seasonal variations on the distributions of *Plasmodium* species and also to compare the intensity of malaria transmission in area with different ecology and seasons in the same region. Findings from this study will offer the baseline genomic data for translating relevant epidemiological information for malaria elimination in the Central region of Ghana.

## Method

### Study design

This cross-sectional study was done in the Central region of Ghana. Using systematic sampling approach, malaria samples were collected from study participants living in both predominantly forested and coastal districts in the region. Additionally, study participants were selected in both dry and rainy seasons.

### Selection of study areas and sampling period

From the study region, five districts were randomly selected for this study. The ratio of forested districts to coastal districts in the Central region is 3:2. Therefore by optimum allocation, three districts were randomly selected from among the forested districts while two districts were selected from among the coastal districts. Forested zone districts involved in this study were Agona Swedru, Assin Central, and Gomoa East while in the coastal zone, the participating districts were Abura-Asebu-Kwamankese and Cape Coast. From these districts, participants with malaria were recruited from the respective district hospitals. This study was carried out, in all the sites concurrently, during the minor rainy and dry seasons (September 2020 –February, 2021). These are the seasons with stable transmission of malaria in the region [17].

### Study population

From each participating district hospital, study participants that were suspected of malaria were included in this study. Fever, headache and chills were the criteria for suspecting malaria. However, participants that have initiated anti-malarial therapy and those that received parenteral fluids before the microscopy report was received were excluded. Additionally, study participants that have ever travelled to any of the south-east Asian and central and northern African countries were excluded from study. More importantly, participant consent and parental assent was key for inclusion.

### Sample size determination and blood sample sampling procedure

From previous study, the prevalence of microscopy detectable malaria was 38.2% (Dakorah et al., 2022). Therefore, using the Cochrane’s formula; n = z^2^p(1-p)/d^2^, where n = sample size, z = confidence level at 95% (standard value of 1.96), d = margin of error at 5% (standard value of 0.05), the sample size was calculated to be 363. Adjusting for microscopy errors of about 23% [18], a minimum of 446 samples were collected.

### Laboratory procedures

#### Blood sample collection, malaria screening and preparation dried blood spots

Blood samples were collected from study participants suspected of malaria. A minimum of 4 mL of whole blood was collected into EDTA tube. Mixed gently to achieve complete anticoagulation. The samples were initially screened for exposure to malaria parasites using the CareStart mRDT (Access Bio, Somerset, USA) and later parasitaemia determined as earlier published [19]. In brief, 6 μL of whole blood were used to prepare thick blood films, air dried, stained with 10% Giemsa for 10 min, and examined using the light microscope. From the microscopy detectable samples, four dried blood spots were made as prescribed by Malaria Genome Laboratory (MGL) of Wellcome Sanger Institute (WSI).

#### *Plasmodium* species identification and determination of multiclonal infections through amplicon sequencing

A 3 mm diameter single-hole paper punch was used to cut the DBS micro-centrifuge tubes from which DNA was extracted using QIAamp DNA Investigator Kit (Qiagen, California, United States) following kit manufacturer’s instructions. A minimum of 5 ng gDNA was used as template for the amplicon sequencing. The specific primers used to detect the five species of the *Plasmodium* (*P*. *falciparum* from *P*. *vivax*, *P*. *knowlesi*, *P*. *malariae* and *P*. *ovale*) has been previously published [12].

#### Selective whole genome amplifications (SWGA) of *Plasmodium* genome

The step-wise method for the SWGA has been published in a separate study [20]. In summary, genome amplification reaction was performed in 50 μL reaction volume containing at least 5 ng of template DNA, 1× BSA, 1 mM dNTPs, 2.5 μM of each amplification primer, 1× Phi29 reaction buffer and 30 units of Phi29 polymerase enzyme (New England Biolabs). Amplification conditions were using step down temperatures from 35°C to 30°C with increasing incubation time from 5 mins to 16 hours. The polymerase enzyme was inactivate at 65°C prior to cooling to 4°C.

#### Illumina amplicon sequencing using HiSeq 2500 sequencer

The Illumina sequencing technology used to sequence *Plasmodium* spp has previously been described [20]. In brief, The SWGA amplicons were cleaned using Ampure XP beads and incubated for 5 min at 25°C. After which the bead/DNA mixture was placed on a magnetic rack to capture the DNA-bound beads. Beads were washed twice with 200 μL of 80% ethanol and the bound DNA eluted with 60 μL of elution buffer. Cleaned amplified DNA products (approx. 0.5–1 μg DNA) were used to prepare DNA library using the NEBNext DNA sample preparation kit (New England Biolabs) for high throughput sequencing.

#### Analysis of sequence data

*P*. *falciparum* sequence reads were demultiplexed and fastq data files generated automatically. Low quality bases were trim with Bioedit v7.2. Each dataset was analysed independently by mapping sequence reads to the *P*. *falciparum* 3D7 reference genome using Burrows-Wheeler Aligner (BWA). SNP analysis was performed on sequenced data targeting SNPs present in the core genome. *P*. *falciparum*, *P*. *vivax*, *P*. *knowlesi*, *P*. *malariae* and *P*. *ovale* were genotyped using differences in the parasite mitochondria (PfMIT:270). The complexity of infection was determined using the COIL (complexity of infection likelihood) computational analysis. COIL analysis is based on the assumption that distinct parasite lineages in complex infections are unrelated. Hence, genotyped loci do not exhibit significant linkage disequilibrium. COIL uses the binomial distribution to estimate the likelihood of a COI level given the prevalence of observed monomorphic or polymorphic genotypes within each sample [21].

### Data analysis

**Fig 1:**
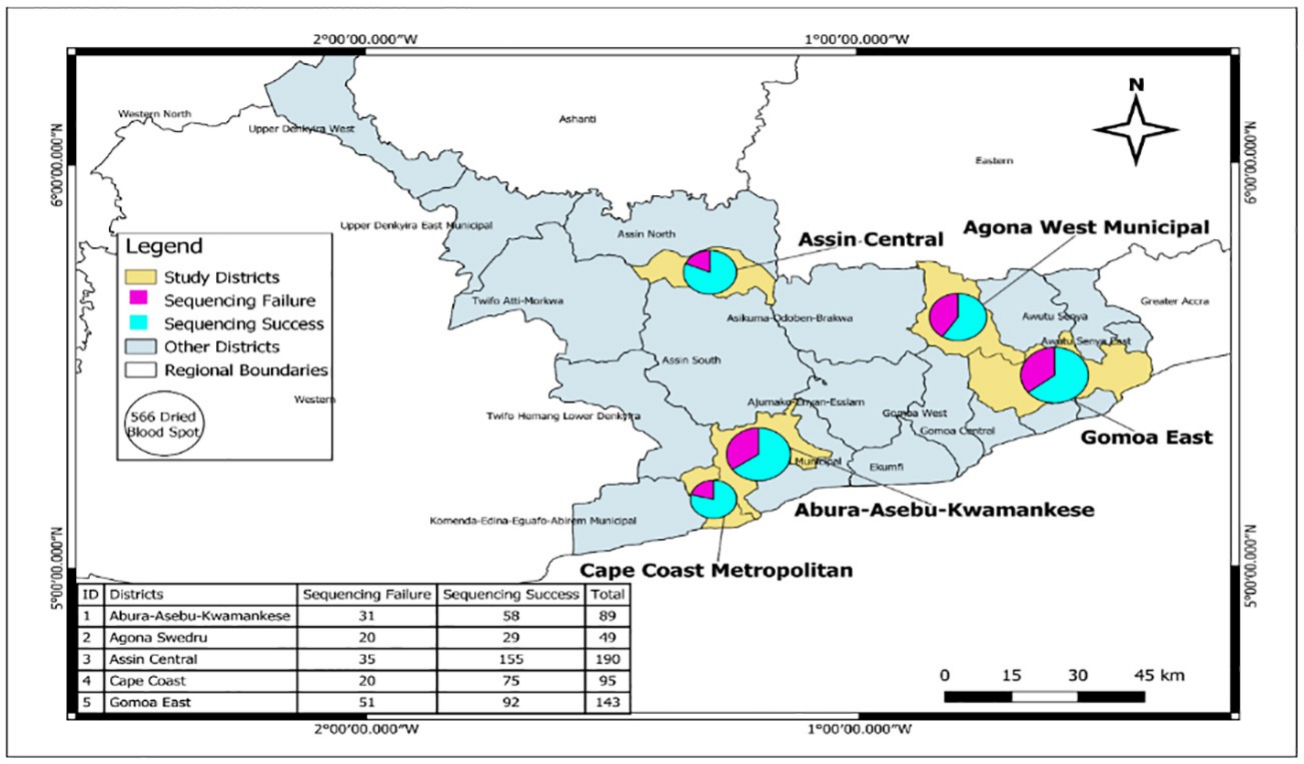
Study distribution of *Plasmodium* genome sequencing success and failure rates. The map was created by the authors using QGIS (Version 3.20.3), QGIS is an open source software hence license was not required.

The prevalence of each parasite species and its distribution across the study sites was presented as percentages. The COI values were expressed as mean ± standard deviation. In binary data, t-test was used to determine the differences in the mean values. The differences in the mean values were assessed using one-way analysis of variance (ANOVA). Chi square was used to test the association between COI and independent variables. The analyses were performed using STATA version 15. In all, p<0.05 was considered statistically significant. Quantum Geographic Information System (QGIS), version 3.20.3 (1), was used to create base layer of study distribution of *Plasmodium* genome sequencing success and failure rates (Fig 1). QGIS was used to manipulate shapefiles. Humanitarian data exchange provided shapefiles of subnational administrative boundaries.

### Ethics approval and consent to participate

Ghana Health Service Ethical Review Committee (GHSERC017/03/20) approved this study. Written consent for participation was sought from each adult participant while written parental assent was obtained for participants less than 19 years.

## Results

### *Plasmodium* genome sequencing success and failure rates

A total of five hundred and sixty six (n = 566) dried blood spots with microscopy detectable parasites were sequenced. Of this number, *Plasmodium* genome was detected in 92.2% (522/566) of the samples while *Plasmodium* genome was not detected in 44 of the 566 samples (7.8%). Overall, 27.7% (157/566) samples failed to be sequenced while the rest (409/566, 72.3%) were successfully sequenced. Most of the samples that were successfully sequenced were obtained from Assin Central (n = 190) while the least were obtained from Agona Swedru (n = 49). It was interesting to observe that 20/49 (~41%) of the samples obtained from Agona Swedru failed to be sequenced (Fig 1).

### Prevalence of *Plasmodium* spp in the study districts related to other variables

**Fig 2:**
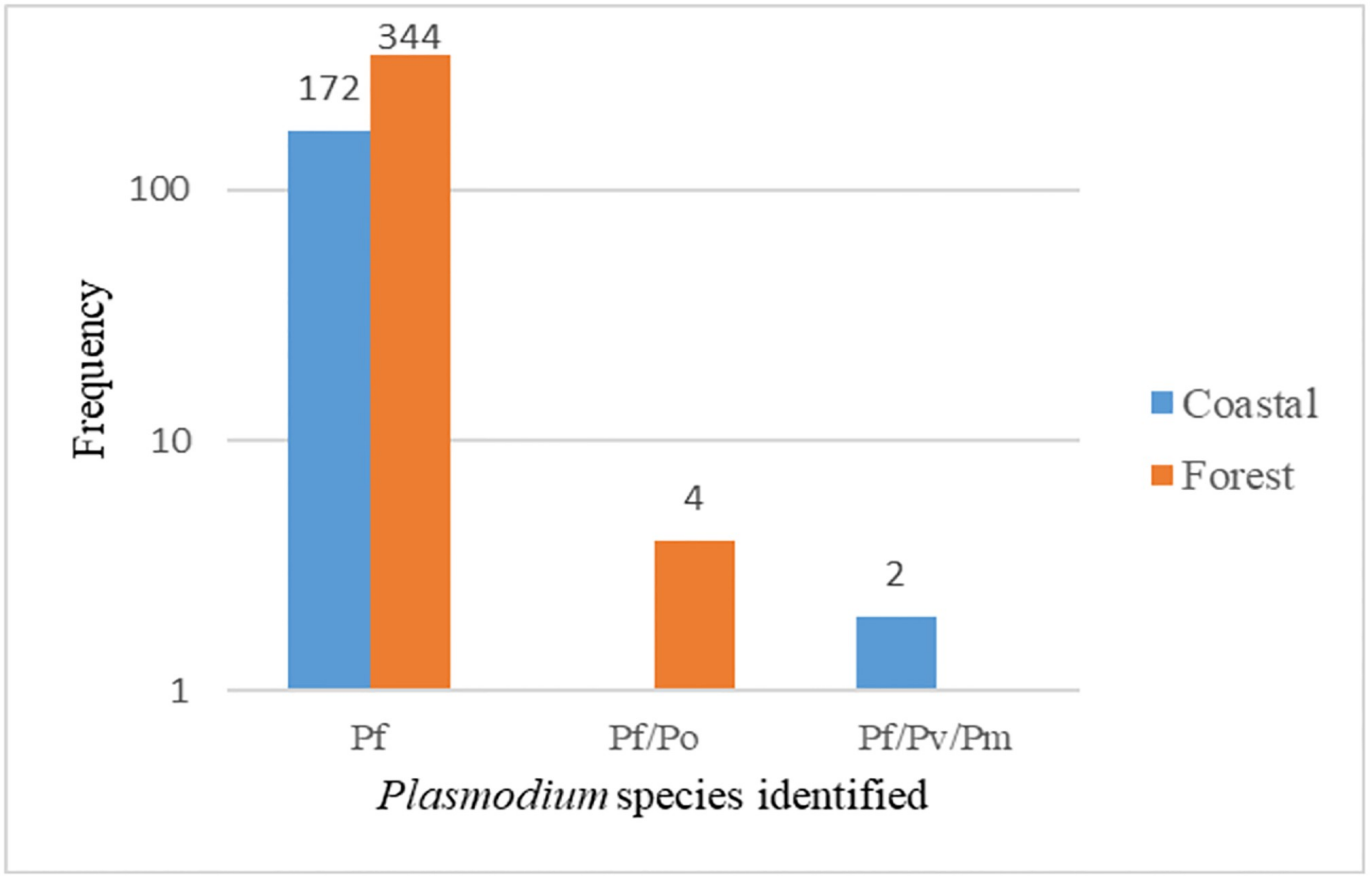
Ecological distribution of the *Plasmodium* species.

**Fig 3:**
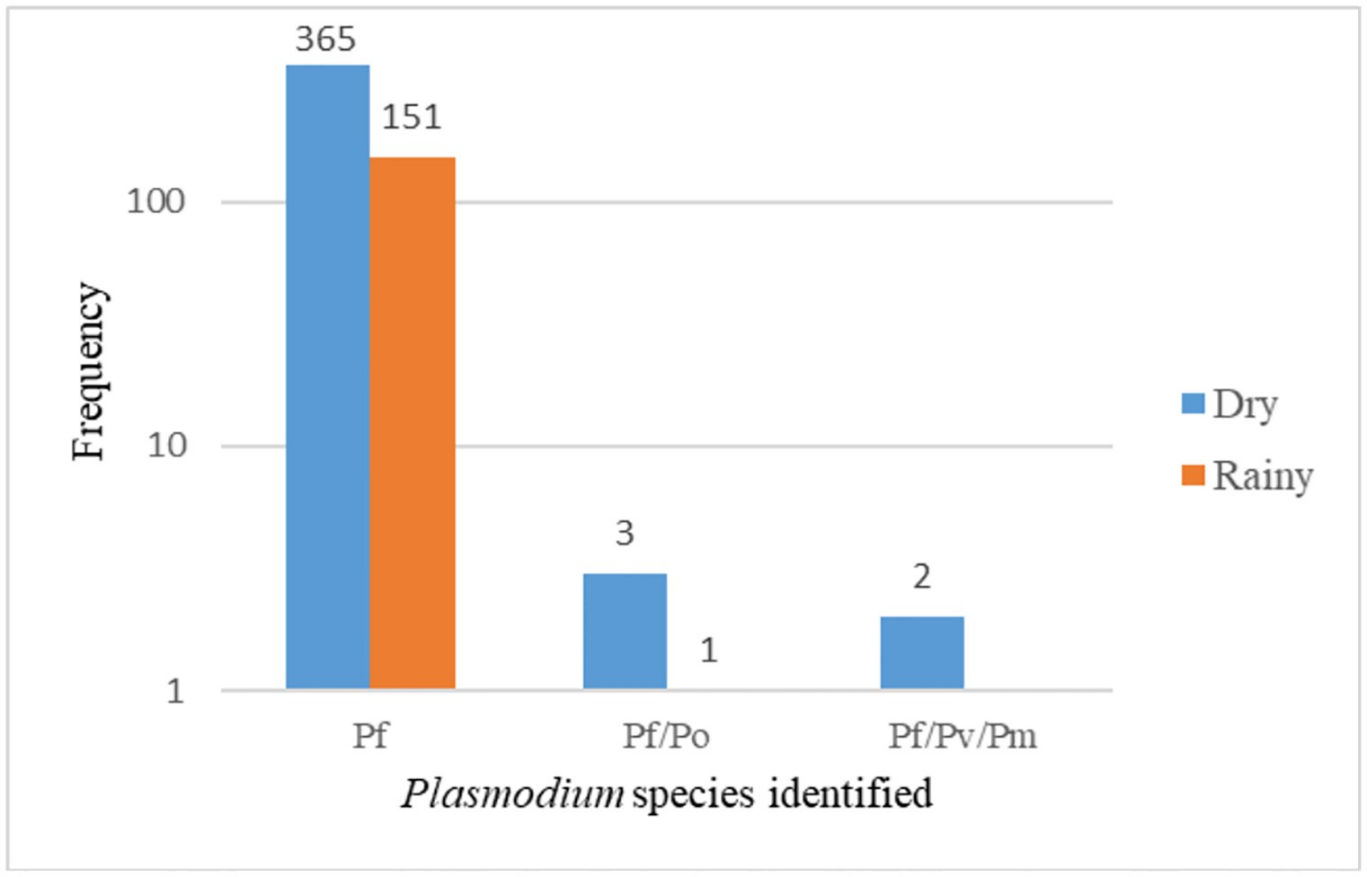
Seasonal distribution of the *Plasmodium* species.

Based on the single nucleotide polymorphism (SNPs) in the *Plasmodium* mitochondria (PfMIT:270), the *Plasmodium* spp were identified. Genomic analysis revealed that, all the samples contained *Plasmodium falciparum*. Of the total, 516/522 (98.8%) of the samples contained *P*. *falciparum* mono-infection while the rest (1.2%) were either *P*. *falciparum/P*. *ovale* (*Pf/Po*) co-infection (n = 4, 0.8%) or *P*. *falciparum/P*. *malariae/P*. *vivax* (*Pf/Pm/Pv*) triple infection (n = 2, 0.4%). All the four *Pf/Po* co-infections were identified in samples from the Assin Central Municipality (two males and two females). The infected participants were aged 1 (male infectee; parasitaemia– 9584 parasites/μL), 2 (male infectee; parasitaemia– 36482 parasites/μL), 16 (female infectee; parasitaemia– 593 parasites/μL) and 38 (female infectee; parasitaemia– 23140 parasites/μL) years. Further, the two *Pf/Pm/Pv* triple infections were identified in male resident in Abura-Asebu-Kwamankese district and Cape Coast Metropolis (Table 1). The infectees were aged 7 (seen in Abura-Asebu-Kwamankese district; parasitaemia; 756 parasites/μL) and 16 (seen in Cape Coast Metropolis; parasitaemia; 1960 parasites/μL) years. Interestingly, all the *Pf/Po* were seen in forested study sites while all the *Pf/Pm/Pv* were seen in the coastal sites. On the other hand, 3 of 4 of the *Pf/Po* were identified during the dry season while one *Pf/Po* co-infection was identified during the minor rains. Regarding the triple infections (*Pf/Pm/Pv*), all the two were identified during the dry season (Figs 2 and 3).

**Table 1:** Prevalence and distribution of *Plasmodium* spp by study sites and gender.

Species	Study districts, n (%)										
	Overall	AAK		ASM		CCM		ACM		GED	
	n = 522	n = 84		n = 38		n = 90		n = 183		n = 127	
		Female	Male	Female	Male	Female	Male	Female	Male	Female	Male
		64 (76.2)	20 (23.8)	24 (63.2)	14 (36.8)	47 (52.2)	43 (47.8)	108 (59.0)	75 (41.0)	79 (62.2)	48 (37.8)
*Pf*	516 (98.8)	64 (100)	19 (95)	24 (100)	14 (100)	47 (100)	42 (97.7)	106 (98.1)	73 (97.3)	79 (100)	48 (100)
*Pf*/*Po*	4 (0.8)	0	0	0	0	0	0	2 (1.9)	2 (2.7)	0	0
*Pf*/*Pv*/*Pm*	2 (0.4)	0	1 (5)	0	0	0	1 (2.3)	0	0	0	0

*Pf*—*P*. *falciparum*, *Po*—*P*. *ovale*, *Pv—P*. *vivax*, *Pm—P*. *malariae*, AAK—Abura-Asebu-Kwamankese District, ASM—Agona Swedru Municipality, CCM—Cape Coast Metropolis, GED—Gomoa East District, ACM—Assin Central Municipality

### Complexity of *P*. *falciparum* infections (COI)

**Fig 4:**
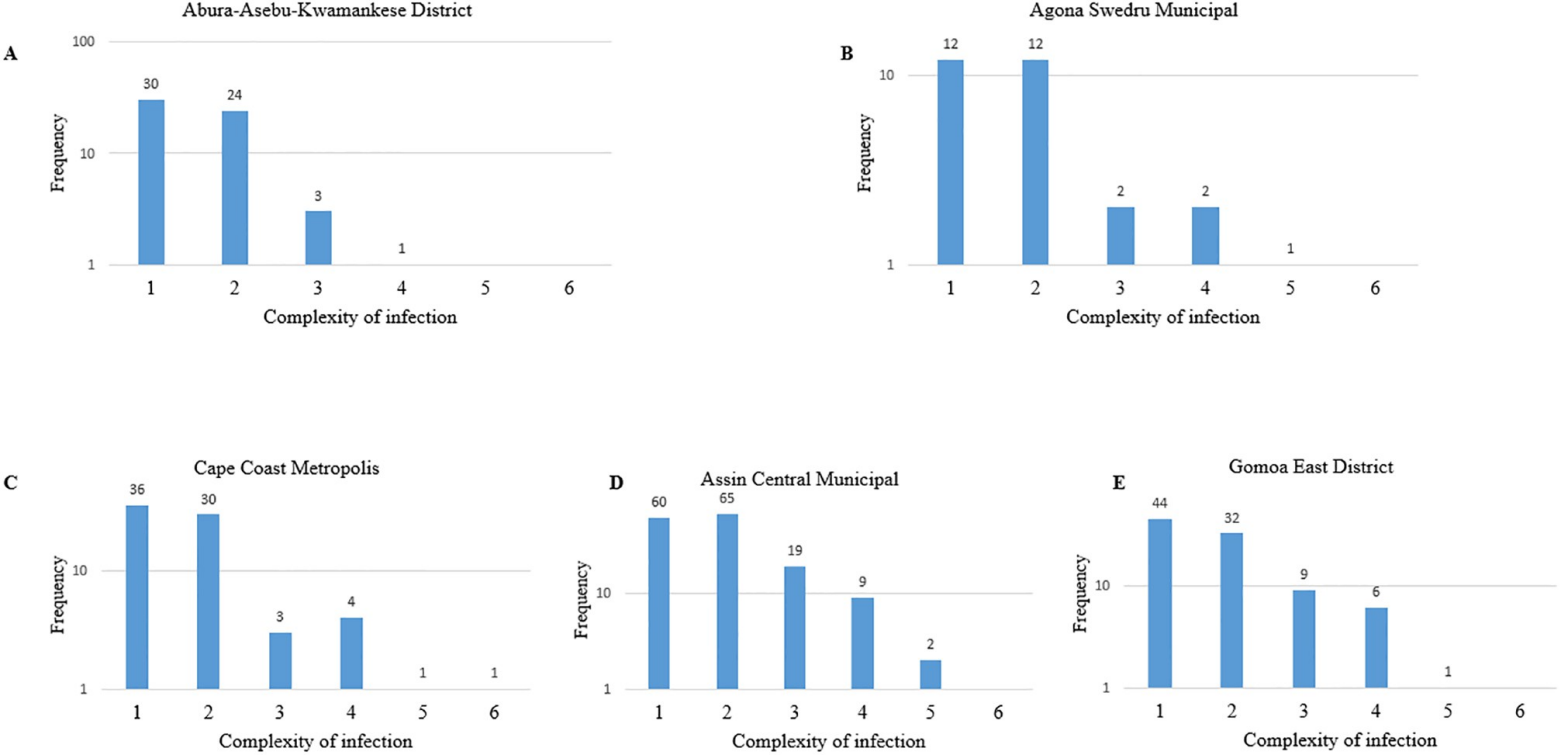
The ranges of complexity of infection (COI) in each study site.

Of the 522 samples successfully genotyped, 409 were successfully sequenced, yielding between 1–6 clones per individual infection. The overall mean COI was 1.78±0.92 (95% CI: 1.55–2.00). Among the study districts, the differences in the COI were not significant (F = 1.4, p = 0.234). Majority (n = 345, 84.5%) of the infections were monoclonal (n = 182, 44.5%) and biclonal (n = 163, 40.0%) whilst the rest (n = 64, 15.6%) were multiclonal (Fig 4). In Abura-Asebu-Kwamankese district *P*. *falciparum* clones up to four were identified (Fig 4A). Up to five clones were observed in Agona Swedru municipal (Fig 4B), Assin Central municipal (Fig 4D) and Gomoa East district (Fig 4E), whereas up to six clones were observed in Cape Coast metropolis (Fig 4C) in one individual.

### Intensity of malaria transmission among age groups and gender

Among the age groups, the mean COI was significantly higher among the 15–19 (mean COI = 2.07), 5–9 (mean COI = 1.98) and > 60 (mean COI = 2.07) year group. The rest of the age groups recorded mean COI < 2. Regression analysis indicated that the transmission of malaria was more that twice among study participants aged 15–19 years (OR = 2.16, p = 0.017) and almost twice among participants aged over 60 years (OR = 1.91, p = 0.021) compared to participants between 20–59 years (reference group). Even those the transmission intensity was higher among participants less than 5 years (OR = 1.17), 5–9 years (OR = 1.48) and 10–14 years (OR = 1.34), the differences were not significant compared to the reference group (Table 2).

**Table 2:** Mean COI by age groups in the study sites.

Age group (years)	Mean COI [95% CI]	F-value; p-value	Adjusted	
			OR [95% CI]	p-value
20–59	1.71 [1.57–1.84]	2.41; 0.0362*	Reference	
< 5	1.79 [1.69–2.18]		1.17[0.99–1.39]	0.071
5–9	1.94 [1.69–2.18]		1.48[1.12–1.95]	0.06
10–14	1.44 [1.16–1.73]		1.34[0.83–2.16]	0.236
15–19	2.07 [1.79–2.36]		2.16[1.80–2.60]	0.017*
≥ 60	2.06 [1.64–2.48]		1.91[1.47–2.24]	0.021*

Regarding the intensity of malaria transmission between the male and female genders, it was found that in all sites, the intensity did not differ between gender except in Gomoa East district where the intensity of malaria transmission was significantly higher in females (mean COI = 1.91±1.02) than in males (mean COI = 1.43±0.50) (p = 0.011) (Table 3).

**Table 3:** Differences in mean COI between genders stratified by study sites.

District	Mean COI ± Standard deviation		t-value (p-value)
	Female	Male	
Abura-Asebu-Kwamankese (n = 58)	1.49±0.55	1.85±0.99	1.70 (0.094)
Agona West (n = 29)	1.94±1.21	1.82±0.75	0.31 (0.758)
Cape Coast (n = 75)	1.85±1.09	1.65±0.92	0.87 (0.382)
Assin Central (n = 155)	1.90±0.90	1.88±0.95	0.09 (0.925)
Gomoa East (n = 92)	1.91±1.02	1.43±0.50	2.60 (0.011)*

### Ecological and seasonal variations on the intensity of malaria transmission in the study districts

Generally, the intensity of malaria transmission across the study sites were similar. That notwithstanding, the number of *P*. *falciparum* clones causing infections in the forested study sites (COI = 1.86) and during the dry season (COI = 1.81) were slightly higher, although not statistically significant (Table 4).

**Table 4:** Ecological and seasonal variations of *P*. *falciparum* clones.

	Mean COI	Standard Deviation	95% Confidence Interval		*P-value*
			Lower limit	Upper limit	
Ecological zones					0.0681
Coastal	1.68	0.88	1.53	1.83	
Forest	1.86	0.94	1.74	1.97	
Seasons					0.8034
Dry	1.81	0.99	1.64	1.98	
Rainy	1.79	0.90	1.68	1.90	

## Discussion

It was found in this study that 98.8% of the study participants who had malaria were infected with *P*. *falciparum* whereas 0.8% and 0.4% were co-infected with *P*. *falciparum*/*P*. *ovale* (*Pf/Po*), and multiple infection of *P*. *falciparum*/*P*. *vivax/ P*. *malariae* (*Pf/Pv/Pm*). The participants were infected with up to 6 genetically distinct clones of *P*. *falciparum*. However, the differences in the mean number of clones did not differ across study sites, and between seasons and ecological zones. In spite of these observations, the risk of being infected with P. falciparum was more than twice and almost among 15–19-year and > 60-year age group compared to participants 20–59 years old. Genomic data described in this study was obtained through whole genome sequencing (WGS). This was the technology available at Wellcome Sanger Institute, Malaria Genome Laboratory, where samples reported in this study were analyzed. Unlike the polymerase chain reactions (PCR) and quantitative PCR that can only detect and quantity *Plasmodium* genome respectively, the WGS had an added advantage of determining the number of parasite clones as well as other single nucleotide polymorphisms.

In Ghana, *Plasmodium falciparum* is the main cause of malaria, causing more than 98% of malaria cases, per a study carried out over a decade ago in the Ahafo area of Ghana, while *P*. *malariae* and *P*. *ovale* shared the rest [22]. In 2017, another study in the Kwahu-South district in the Eastern region of Ghana reported the prevalence of *P*. *falciparum* to be 87.3% whereas *P*. *malariae* was 12.7% [23]. It is interesting to note that in 2020, a study done in the Ashanti region of Ghana reported a further decline in the proportions of *P*. *falciparum* (68.0%) in relation to *P*. *malariae* (8.0%) and *P*. *ovale* (9.0%) with the rest being mix-infections [24]. Even though the study settings and the period those studies were conducted differed, the variations in the data reported were striking. The first study that reported the *Plasmodium* species prevalence in the Central region of Ghana observed a further decline in the *P*. *falciparum* population (61.4% in 2017 and 57.9% in 2019) while *P*. *malariae* and *P*. *ovale* populations increased significantly from 2.4% (2017) to 20.7% (2019), and 0% (2017) to 9.1% (2019) [25]. It was observed in this study that malaria in the forest zones were twice as high as prevalence observed in the coastal zones. A similar observation was seen between dry and rainy season, where malaria in the dry season was more than twice the prevalence in the rainy season. Whereas forest ecology is conducive to support *Plasmodium* spp survival and longevity, during the dry season, pockets of stagnant water are cleaner and calmer enough to complete the life cycle of the mosquito.

Considering the previous data reporting the decline of the prevalence of *P*. *falciparum* in study participants, it was surprising to observe that the prevalence of *P*. *falciparum* in this study was 98.8% and that of *Pf/Po* and *Pf/Pv/Pm* were unexpectedly low (0.8% and 0.4% respectively). In 2020, a study recorded similar prevalence of *P*. *falciparum* (95.9%) in the Central region while non-falciparum species (*P*. *malariae* only) was seen at 4.1% [26]. It is noteworthy that in two of the study districts that share border that is Gomoa East and Agona Swedru, 100% prevalence of *P*. *falciparum* was observed. Due to the fact that *P*. *falciparum* is more virulent compared to the other *Plasmodium* spp [27], it was not surprising that *P*. *falciparum* was responsible for majority of the disease. Another finding worth nothing was that, all the other non-falciparum species identified, co-infected with the falciparum species. However, this is not new in Ghana, since a previous study has recorded almost the same prevalence (0.7%) of *Pf/Po* in a nationwide study that analysed 5260 samples [28]. The other mixed-infection that was found in this study was *Pf/Pv/Pm*. To the best of our knowledge this is the first time *P*. *vivax* is being identified in Ghana. However, several cases of *P*. *vivax* have been identified in various sites in Nigeria [29–32], Cameroon [33, 34], Benin [35] and Senegal [36]. In this current study, the *Pf/Pv/Pm* mixed-infections were seen in two males (7 and 16 years), from two different but contiguous districts (Abura-Asebu-Kwamankese district and Cape Coast metropolis). These individuals do not have any foreign travel history. It is known that *P*. *vivax* cause infection in individuals with Duffy erythrocytes, however *P*. *vivax* infection in Africans without Duffy erythrocytes has also been reported [37]. Considering the fact high sensitive Illumina sequencing technique was used to genotype the *Plasmodium* based on the single nucleotide polymorphism in the *Plasmodium* mitochondria [38], the identification of *P*. *vivax* cannot be taken for granted. The Central region of Ghana has several tourist sites. Five major sites that receive tens of thousands visitors from across the globe are the Cape Coast Castle (at Cape Coast), the Elmina Castle (a UNESCO World Heritage Site at Elmina), the Kakum National Park (north of Cape Coast), the Posuban Shrines (found in Mankessim, Elmina and Anomabu) and the International Stingless Bee Centre (found in Odumase Abrafo). In 2021, 86040, 47930, 36300 tourist from all over the world visited the Kakum National Park, Cape Coast Castel and Elimina Castle alone [39]. These tourists can import *P*. *vivax* into the sites visited.

Analysis of the whole genome sequences of the *P*. *falciparum* made it possible to identify the different parasite clones in each sample, using the complexity of infection (COI) likelihood Bayesian modelling. Whereas previous studies [16, 40] reported a direct relationship between COI and transmission intensity of malaria, another study [41] reported otherwise. However, considering the fact COI is increased in instances of independent bites of mosquitoes carrying the sporozoites or a single mosquito bite carrying a genetically diverse sporozoite [15], we believe that a relationship exit, although pre-existing anti-malaria immunity, which is short-lived [42, 43], could affect this relationship. The current study demonstrated that there is clonal expansion of *P*. *falciparum* infections in the Central region with 55.5% of the study participants having between 2–6, similar to the 56% prevalence reported by Tandoh et al. [44]. Furthermore, the prevalence of polyclonal infection also corroborates with findings on parasite diversity in other parts of Ghana, which concluded that *P*. *falciparum* parasite infections in southern Ghana were diverse after analyzing malaria-positive samples from Obom (in the Greater Accra region) and Asutsuare (in the Easter region) [45].

The overall mean complexity of infection (COI) (COI is the number of genetically distinct parasites that infect a patient, at the same time) of the current study (1.78±0.92) was slightly lower compared with other studies elsewhere; Congo– 2.64 [46] and 2.38 when comparing AMA1 amino acid frequencies [47–49]. This study found most of the infections to be monoclonal (44.5%) and biclonal (40.0%). Infections with more than two clones were 15.6%. It was also noted that intensity of malaria transmission was similar among study sites and in both male and female gender in all study sites, except in Gomoa East where transmission of malaria is expected to be higher in females than males. Additionally, transmission of malaria was higher in participants aged 5–9 years, 15–19 years and > 60 years. Aside that, it could be concluded that the transmission intensity of malaria in the study sites was similar. The ecology and rainfall patterns support this observation. Even though minority districts in the region are coastal, a section of those districts have forest, making the region predominantly forest region. It has been established that forest ecology influence transmission of malaria. This is because, forests provide conditions such as vegetation cover, temperature, rainfall and humidity conditions that are conducive to distribution and survival of malaria vectors [50]. At the coastal districts in the Central region, coconut plantation lines the coast [51]. The endocarp of an indiscriminately discarded eaten coconut can collect rain water which could serves as breeding places for mosquitoes. Additionally, the outdoor activity of the fishing business, at both day and night, also exposes the fisher folks to the mosquito bites. Additionally, a study in Cameroon reported that four species of *Anopheles*, namely, *Anopheles gambiae*, *Anopheles arabiensis Anopheles funestus*, *Anopheles nili* and *Anopheles moucheti* are very prevalent along coastal zones and they have the ability to resist being swept away by the sea breeze at resting positions [52].

The Gomoa East district has three major markets located in Gomoa Buduburam, Gomoa Nyanyano Kakraba and Gomoa Dominase Junction [53] where there are economic activities, both day and night. Since females dominate trading activities in Ghana [54], the observation that transmission intensity in females were high was not surprising. Further, observation that malaria transmission was high in individuals aged 5–9 years, 15–19 years and > 60 years, is of both clinical and epidemiological importance. Individuals in these age groups especially, 5–9 and 15–19 years, sleep less often in insecticide treated nets [55] and frequently engage in prolong outdoor activities [56] including at night [57]. This exposes these groups to multiple mosquito bites. Similar factors explains the high transmission of malaria in the over 60 year group together with reduced immunity [58].

### Limitation

Of the 566 samples submitted for sequencing, sequencing failed for 201 (35.5%) samples. Excluding these samples from analysis could affect the frequencies and associated factors reported in this study.

## Conclusion

This study reports disproportionately high prevalence of *P*. *falciparum* (98.8%) and low prevalence of *P*. *falciparum/P*. *ovale* (0.8%) and *P*. *falciparum/P*. *vivax/P*. *malariae* (0.4%) mixed infections. This study reports for the first time *P*. *vivax* in Ghana, albeit at very low frequency. Malaria transmission was found to be similar in the study sites, except among study participants aged 15–19 years and > 60 years where malaria tramsnsiion was found to be higher compared to 20–59 year age group. This study calls for active surveillance for *P*. *vivax* in other parts of Ghana to confirm the parasite as an emerging pathogen in the country. Additionally, enhanced malaria control measures must be instituted in the Central region of Ghana especially among 15–19-year and > 60-years, since malaria transmission was higher among them.

## Supporting information

S1 Data The detailed study participant data comprising demographic information, malaria screening outcome and *Plasmodium* genetic information. (XLSX)
